# Physiological response of roe deer (*Capreolus capreolus*) during stalking hunts depending on age

**DOI:** 10.1186/s12917-023-03833-8

**Published:** 2023-12-09

**Authors:** Katarzyna Dziki-Michalska, Katarzyna Tajchman, Sylwester Kowalik

**Affiliations:** 1 Department of Animal Ethology and Wildlife Management, Faculty of Animal Sciences and Bioeconomy, University of Life Sciences in Lublin, Akademicka 13, Lublin, 20-950 Poland; 2 Department of Animal Physiology, Faculty of Veterinary Medicine, University of Life Sciences in Lublin, Akademicka 12, Lublin, 20-033 Poland

**Keywords:** *Capreolus capreolus*, Rapid stress response, Hormone response, Blood chemistry indices

## Abstract

**Background:**

The European roe deer (*Capreolus capreolus*) is a species particularly reactive to all kinds of negative stimuli. Hunting activity is one of the most potent stressors that disturbs the welfare of wild animals. During stress, various endocrine responses are elicited to improve the physical performance of the affected individual. A commonly assessed hormone for overcoming stressful situations is cortisol (CORT). In this study, plasma CORT levels in roe deer were assessed during the season of the most intense stalking hunts in Poland (summer vs. late autumn), the sex of the harvested animals (males vs. females), and age of harvest animal. In addition, the health status of the roe doe was evaluated on the basis of selected indices of blood chemistry, which could be associated with circulating cortisol levels.

**Results:**

The mean cortisol levels were 58.066 ng/ml in the male group (summer) and 27.694 ng/ml in the female group (late autumn). Higher CORT levels were associated with a significantly lower of total cholesterol, lactate dehydrogenase, and uric acid (*p* < 0.05). Moreover, the mean concentration of uric acid was negatively correlated with the level of CORT in the male and female groups (*p* < 0.05). Together with the increase in mean CORT level, the HDL cholesterol of all the tested animals increased significantly (*p* < 0.05).

**Conclusions:**

Higher CORT in males during the summer than in females during the late autumn were most likely due to the arousal with the mating season. The level of CORT increased with the animals’ age. Uric acid and age are both predictors of roe deer’s serum CORT level.

## Background

The European roe deer population is large (over 900.000 individuals in the 2021/22 season) [1], and it is a popular species for hunting in Poland (over 200.000 individuals shot in the 2021/22 season) [1]. In the face of situations perceived as a threat, its behavioral response includes increased vigilance, immobility, and escape. Vertebrates are sensitive to all kinds of stress stimuli, to which they show a neuroendocrine response. Reimosers’ [2] research showed that the reactions of roe deer towards such stimuli were brief and strong, whereas red deer showed less obvious but persisting reactions. In addition, roe deer show greater impetuousness compared to other cervidae [3, 4]. Negative events, including both endogenous and exogeneous stimuli, can active an endocrine response, which includes the release of corticosteroid hormones from the adrenal cortex into the bloodstream. This enables the body to take quick action aimed at eliminating and avoiding the impact of the stimulus [5–7]. It is well-known that a short-term stressor has a mobilizing effect (e.g., escaping a predator, moving toward a refuge) on the body and is essential for survival in times of danger [8]. Cortisol (CORT) is one of the most common stress markers in animals. There are many ways to measure this hormone, including in saliva, blood, feces, urine, or hair [9]. However, with hair we can characterize only long-term stress [10, 11], and in feces only an integrated measurement over a certain amount of time of stressor reaction can be acquired; for roe deer, this can be 18 h [12] or 12 h [13]. The analysis of saliva and blood allows us to obtain reliable information to determine worsening short-term stress. However, collecting saliva from free-living animals is problematic because the effect of immobilization would likely increase the stress response [14–16]. A good method is rapid postmortem blood sampling, in which the CORT level reflects the severity of stress [7, 17–19]. The level of CORT in the bloodstream directly affects the blood’s biochemical parameters, and these, in turn, reflect the animal’s welfare state. Previous studies confirmed that exposure of an organism to acute stress negatively affects the immune system [20, 21], which in turn increases its susceptibility to various diseases [22]. Hunting is widely recognized as one of the most potent factors which causes repeated and long-term stress in animals [12]. Consequently, all such negative stimulators can affect their growth and development and thus transpose into their individual reproductive success [18–20, 23–25]. It is therefore vital that the subject of welfare is no longer considered only in the context of farm animals, but also applied to free-living animals, especially in relation to rationally conducted hunting management [17].

The results of the research that has been conducted on the seasonality of changes in CORT levels are divergent. In cervids, increased CORT levels are typical in the winter [15, 24, 26–29]. However, recent reports indicate an increased concentration of this hormone in deer during the growth and mineralization of antlers, i.e., in early spring and late summer [30–32]. It is likely related to species differences and sensitivity to climatic factors [26]. The effects of sex and age on CORT levels and blood biochemistry remain unresolved. Comparative studies do not show a statistically significant difference in CORT levels between female and male deer [15, 26, 33–35]. It is presumed that negative environmental stimuli are more important for CORT fluctuations [17]. Moreover, some pathological conditions, such as the clinical and subclinical states of many diseases, affect the levels of CORT in ruminants [36–39]. Similarly, as shown previously, the differences in breeding conditions of farmed deer significantly influence a number of blood constituents and hormones [36]. Importantly, in all cases, the most commonly used methods of their detection are blood tests, e.g., the level of selected morphological and biochemical parameters.

Collective or individual hunting, as well as frequent presence of hunters on the hunting grounds, directly influences the intensity of the hormonal reaction in cervids [12], which may have a negative impact on their welfare. Bateson and Bradshaw [40] showed that hunting arrows cause physiological effects in red deer (*Cervus elaphus*), such as an increase in the level of markers of skeletal muscle damage and changes in the concentration of β-endorphin.

Active hunting events using hounds or involving large numbers of hunters generate a more intense endocrine reactions and metabolic demands on the body.Current research clearly shows that stalking is the least stressful deer hunting technique, where one well-aimed shot can result in the quick death of the animal [12, 17, 41], but there are few reports on its impact on roe deer, the species that is the least resistant to stress among game animals.

This research aimed to assess plasma CORT levels in roe deer (*Capreolus capreolus*) depending on: (1) the season of the most intense stalking hunts in Poland (summer vs. late autumn); (2) related to the hunting season sex of the harvested animals (males vs. females) related to the hunting season; and (3) the age of the harvested animals. In addition, the fourth aim was to evaluate several indices blood chemistry, which reflect the animal’s general health and may be associated with the circulating level of cortisol.

## Results

Males had a mean carcass mass of 18.563 kg and were on average 2 years older than females, whose average carcass mass was 16.895. The mean CORT level in males was 58.066 ng/mL, approximately 2 times higher than in females (27.694 ng/mL), but the lactate dehydrogenase (LDH) level was higher in females (6203.895 U/L) than in males (1181.250 U/L). There were no differences in the other blood chemical values between males and females, except for alanine aminotransferase (ALT) being higher in females (342.126 U/L) than in males (236.487 U/L) (Table 1).

**Table 1:** Carcass mass, age, and blood chemistry indicesmales and females roe deer

Indicator	Males (n = 16)		Females (n = 19)	
	M	SD	M	SD
CM [kg]	18.563	1.364	16.895	2.998
A [years]	5.813	2.104	3.632	1.300
CORT [ng/ml]	58.066	21.968	27.694	17.470
TCHOL [mg/dL]	61.750	12.741	65.000	15.173
HDLC [mg/dL]	21.063	7.007	17.074	8.528
TRIG [mg/dL]	185.938	71.207	200.474	58.229
LDH [U/L]	1181.250	878.189	6203.895	2402.976
UREA [mg/dL]	75.450	13.293	84.684	27.909
ALT [U/L]	236.487	180.672	342.126	232.615
URIC [mg/dL]	6.137	3.911	7.647	3.753
ALP [U/L]	135.163	173.385	141.416	86.453
TP [g/dL]	8.063	1.0626	8.053	1.079
HSA [g/dL]	3.750	0.4472	3.632	0.496

CM- carcass mass, A- age, CORT- cortisol, TCHOL- total cholesterol, HDLC- cholesterol HDL, TRIG- triglycerides, LDH- lactate dehydrogenase, UREA- urea, ALT- alanine aminotransferase, URIC- uric acid, ALP- alkaline phosphatase, TP- total protein, HSA- albumin, M-mean, SD- standard deviation

The strength of the association between CORT and the blood chemistry was examined for all deer considering the data from both females and males. Mean total cholesterol (TCHOL) and LDH decreased significantly with increased CORT in plasma from all roe deer (respectively, rs = -0.416, *p* = 0.012 and rs = -0.381, *p* = 0.024), but mean cholesterol HDL (HDLC) increased significantly (rs = 0.445, *p* = 0.007). The mean uric acid (URIC) concentration was significantly negatively correlated with CORT levels in males (rs = -0.767, *p* < 0.001) and females (rs = -0.472, *p* = 0.040), and in all animals tested together (rs = -0.618, *p* < 0.001) (Table 2).

**Table 2:** Association of cortisol and blood biochemistry in roe deer

Analyzed parameters	CORT					
	**Males** (n = 16)		**Females** (n = 19)		**All animals** (n = 35)	
	**rs**	***p***	**rs**	***p***	**rs**	***p***
TCHOL [mg/dL]	-0.446	0.082	-0.356	0.134	-0.416	0.012*
HDLC [mg/dL]	0.408	0.116	0.144	0.554	0.445	0.007*
TRIG [mg/dL]	-0.312	0.239	0.173	0.477	-0.079	0.650
LDH [U/L]	0.038	0.888	0.372	0.117	-0.381	0.024*
UREA [mg/dL]	-0.100	0.712	0.307	0.201	-0.005	0.973
ALT [U/L]	0.232	0.386	0.307	0.201	0.170	0.328
URIC [mg/dL]	-0.767	< 0.001*	-0.472	0.040*	-0.618	< 0.001*
ALP [U/L]	-0.382	0.143	0.008	0.971	-0.270	0.115
TP [g/dL]	-0.199	0.458	-0.367	0.121	-0.222	0.199
HSA [g/dL]	-0.375	0.151	-0.239	0.324	-0.152	0.382

CORT- cortisol, TCHOL- total cholesterol, HDLC- cholesterol HDL, TRIG- triglycerides, LDH- lactate dehydrogenase, UREA- urea, ALT- alanine aminotransferase, URIC- uric acid, ALP- alkaline phosphatase, TP- total protein, HSA- albumin, rs- Spearman rank-order correlations, * statistically significant values at *p* < 0.05

The mean level of LDH decreased with increasing age (rs = -0.449, *p* = 0.006) in the researched animals (Table 3).

**Table 3:** Lack of association of the levels of blood chemistry indices with carcass mass and age of roe deer in total

Analyzed parameters	Carcass mass		Age	
	rs	***p***	rs	***p***
CORT [ng/ml]	-0.010	0.953	0.329	0.053
TCHOL [mg/dL]	0.099	0.572	-0.108	0.534
HDLC [mg/dL]	0.064	0.712	0.142	0.414
TRIG [mg/dL]	-0.231	0.181	-0.326	0.055
LDH [U/L]	-0.328	0.054	-0.449	0.006*
UREA [mg/dL]	0.106	0.542	-0.103	0.553
ALT [U/L]	-0.025	0.886	-0.028	0.872
URIC [mg/dL]	0.097	0.575	-0.156	0.368
ALP [U/L]	-0.274	0.111	-0.184	0.288
TP [g/dL]	0.161	0.355	0.035	0.838
HSA [g/dL]	0.071	0.685	-0.065	0.707

CORT- cortisol, TCHOL- total cholesterol, HDLC- cholesterol HDL, TRIG- triglycerides, LDH- lactate dehydrogenase, UREA- urea, ALT- alanine aminotransferase, URIC- uric acid, ALP- alkaline phosphatase, TP- total protein, HSA- albumin, rs- Spearman rank-order correlations, * statistically significant values at *p* < 0.05

There was no significant relationship between blood chemistry values, carcass mass and age in the male group (Table 4).

**Table 4:** Association of the levels of blood chemistry indices and carcass mass and age of males

Analyzed parameters	Carcass mass		Age		
	rs	***p***	rs	***p***	
CORT [ng/ml]	-0.229	0.393	0.056	0.834	
TCHOL [mg/dL]	0.161	0.549	0.202		0.452
HDLCHOL [mg/dL]	-0.077	0.775	-0.190		0.480
TRIG [mg/dL]	-0.112	0.678	-0.251		0.346
LDH [U/L]	-0.161	0.551	0.208		0.438
UREA [mg/dL]	0.441	0.087	0.380		0.146
ALT [U/L]	-0.406	0.118	0.091		0.737
URIC [mg/dL]	0.112	0.678	0.148		0.586
ALP [U/L]	0.364	0.165	0.146		0.589
TP [g/dL]	-0.134	0.620	0.147		0.584
HSA [g/dL]	-0.145	0.591	-0.126		0.639

CORT- cortisol, TCHOL- total cholesterol, HDLC- cholesterol HDL, TRIG- triglycerides, LDH- lactate dehydrogenase, UREA- urea, ALT- alanine aminotransferase, URIC- uric acid, ALP- alkaline phosphatase, TP- total protein, HSA- albumin, rs- Spearman rank-order correlations, * statistically significant values at *p* < 0.05

In the group of females, a significant decrease in mean alkaline phosphatase (ALP) concentration was noted with increased carcass mass (rs = -0.576, *p* = 0.009) (Table 5).

**Table 5:** Relationship between blood chemistry indices, carcass mass, and age of females

Analyzed parameters	Carcass mass			Age	
	rs	***p***		rs	***p***
CORT [ng/ml]	-0.253	0.295		-0.052	0.829
TCHOL [mg/dL]	0.124	0.611		-0.232	0.339
HDLC [mg/dL]	-0.069	0.777		0.067	0.786
TRIG [mg/dL]	-0.281	0.244		-0.317	0.186
LDH [U/L]	-0.108	0.660		-0.153	0.532
UREA [mg/dL]	0.094	0.700		-0.290	0.227
ALT [U/L]	0.254	0.292		0.222	0.359
URIC [mg/dL]	0.227	0.350		-0.116	0.635
ALP [U/L]	-0.576	0.009*		-0.431	0.066
TP [g/dL]	0.288		0.232	-0.003	0.987
HSA [g/dL]	0.101		0.680	-0.095	0.699

CORT- cortisol, TCHOL- total cholesterol, HDLC- cholesterol HDL, TRIG- triglycerides, LDH- lactate dehydrogenase, UREA- urea, ALT- alanine aminotransferase, URIC- uric acid, ALP- alkaline phosphatase, TP- total protein, HSA- albumin, rs- Spearman rank-order correlations, * statistically significant values at *p* < 0.05

In order to determine the influence of selected variables on the CORT concentration, a regression analysis was performed (Table 6). The resulting model was found to be a good fit to the data (F = 13.024, *p* < 0.001). Statistically significant predictors in the model were both URIC (β = -0.547, *p* < 0.001) and age (A) (β = 0.376, *p* = 0.007). The higher the URIC value, the lower the CORT level (assuming that the second variable did not change) and the CORT level increased with increasing A (assuming that the second variable did not change). Such an analysis can be treated as a predictive model. If the URIC concentration increases by one unit, the CORT concentration decreases by 3.511 units (assuming the second variable remains the same). In addition, if age increases by one unit, the CORT value increases by 4.608 units (assuming the other variable remains the same).

**Table 6:** The influence of selected predictors on the level of cortisol in roe deer

Chosen predictors	CORT *R*^2^ skor. = 0.414 *F* = 13.024 *p* < 0.001			
	*B*	*β*	*t*	*p*
Word free	44.678		4.427	< 0.001
URIC	-3.511	-0.547	-4.164	< 0.001*
A	4.608	0.376	2.865	0.007*

F, R^2^, *p*, *B*, *β* – linear regression analysis coefficients, CORT- cortisol, URIC – uric acid, A- age

## Discussion

### Season of the stalking hunts and sex of the roe deer

In Poland, the most intense roe deer stalking hunting period is in August and November. Due to the Polish Hunting Law, the sex of hunted animals is dependent on the season because male deer may be hunted in summer only, while females may only be hunted in autumn. Season and sex will certainly have an effect on serum CORT levels in animals. The higher level of CORT in males obtained in summer compared to females in autumn is probably due to the occurrence of estrus and the ensuing increased activity in defending their territory and following females, because earlier studies have not shown a relationship between the level of CORT and the sex of cervids [15, 17, 26, 33–35]. On the other hand, it was shown that it was in the period of reproductive quiescence, during which new antler growth and rapid weight gain occur, that higher mean plasma CORT levels were obtained [41]. Some authors [15, 26, 33, 35] have shown that high CORT levels are typical of cervidae in the late autumn and winter. However, the biology of roe deer differs from other species of cervids, because their antlers grow in winter and their mating season occurs in summer, which can both affect CORT levels. Results of studies on roe deer have been similar to those obtained in studies on *Rangifer tarandus* [41], *Axis axis* [42], and *Ozotoceros bezoarticus* [43]. However, Gentsch et al. [17] noted lower mean CORT values in their research on roe deer harvested by stalking hunts. Moreover, the same author [17] found in non-stressed roe deer (13.63 nmol/L ± 2.43 as reference values) the levels of CORT were 13 times lower compared to males and 6 times lower compared to females. Male CORT level was similar to roe deer harvested by battue, but female CORT value was higher than roe deer harvested by stalking hunts but lower than roe deer harvested by hunting with dogs [17]. On the other hand, the observed CORT levels in the study by Montané et al. [44] in *Capreolus capreolus* under capture stress were similar to females in our study. However, it should be emphasized that the level of CORT also depends on the characteristics of the behavior of individual animals. Shy or reactive individuals generally respond by expressing higher hypothalamic–pituitary–adrenal axis reactivity (i.e., a higher plasma corticosterone response and body temperature) but lower testosterone activity, characteristic of individuals with low activity and low aggression levels and that are less willing to take risks [3, 45]. Often, the reaction to stress is also a trait related to species, physiological state, living environment, or personality [46]. In studies on roe deer, the range and variability of samples were large, which suggests that the reactivity of a given individual, i.e., individual resistance to stress and the duration of time the subject was exposed to the stimulus, was responsible for the level of the hormone release and may also be an individual trait.

### Blood chemistry indices vs. cortisol level

It is known that stress has a direct impact on animal health, which can be reflected in the levels of blood chemistry measures [46]. In addition, the level of CORT tested also depends on the general state of health, as previously mentioned. The biochemical parameters results obtained by us and the veterinarian’s postmortem inspection did not indicate any pathological conditions in the researched roe deer. When assuming as reference the values obtained from roe deer harvested in the same area but outside the hunting season males obtained during stalking hunts showed 16% higher TCHOL, 11% UREA, 70% ALT and 27% total protein (TP); while in females higher by 20% TCHOL, 20% UREA, 79% ALT and 27% TP [47]. Mean level LDH and TP in the studied roe deer were higher also than those obtained in the study by Montané et al. [44] in *Capreolus capreolus* under capture stress. Mean ALT concentration was lower but urea was similar to roe deer without the effects of sedatives [44, 46]. The concentration of ALP was comparable to that reported by Žele and Vengušt [46] in shot roe deer and by Montané et al. [44] in captured roe deer. Mean serum albumin (HAS) concentration was slightly higher than the values reported by Ursache et al. [48] in captured deer and by Pav et al. [49] and Žele and Vengušt [46] in shot roe deer. The obtained mean values of blood chemistry in roe deer were noticeably higher than in *Alces alces* [50] or *Axis axis* [33], but lower than in *Cervus elaphus* [51]. Variation in these measures may result not only from changes in blood cortisol level, but also from a general sensitivity and the influence of the climate where the tested animals live [12]. These conditions can impact blood chemistry and metabolic pathways, undermining immunological vigor and increasing the risk for infectious disease. Therefore, both a deficiency and an excess of CORT have negative effects on the physiological condition of animals [52, 53].

In studies on roe deer, a negative correlation between TCHOL and CORT was demonstrated, while in all animals tested, the HDLC concentration was high. High cholesterol levels in cervids may lead to cardiovascular diseases [54]. Moreover, TCHOL has a crucial role in cellular and intracellular membranes, which might influence cellular functions in key organs, including the brain. It has been observed that TCHOL level might influence serotonin function [55]. Steroid hormones, including testosterone, progesterone, and estrogens, are also synthesized from cholesterol at the biochemical level [56, 57], which in turn is closely related to reproductive success and the formation of antlers in males [58], and to maintaining pregnancy in females [56]. Moreover, high glucocorticoid level is an important factor in stimulating bone tissue resorption and inhibiting the osteogenesis process [59, 60]. However, it should be emphasized that, for example, liver enzymes can be considered to be acute phase reactants and the levels of some lipids, such as triglycerides, also can change quickly.

The influence of various factors on the fluctuations of the TCHOL and URIC levels in the blood plasma of animals are not fully understood. It is known that serum concentrations of TCHOL are affected by emotional stressors, causing its concentration to increase significantly in the blood. In contrast, serum TCHOL may be decreased by depressive mood or aggression. A change of serum URIC has also been intensively studied as a factor that plays an important role as a stress marker [61, 62].

The high LDH concentration in the plasma of tested roe deer should not be surprising, because LDH is widely used as a marker to diagnose the cause and the site of tissue damage, and the animals were hunted. Previously, this parameter has also been used as a diagnostic criterion when monitoring myocardial infarction [63]. LDH is one of the most specific markers of skeletal muscle damage in animals; therefore, an increase in LDH activity in roe deer plasma may be a useful in vivo marker of post-shooting muscle damage [47, 64]. The activity of ALT, aspartate aminotransferase and LDH muscle enzymes has been reported to increase during capture and handling operations in stressed wild ungulates and animals suffering from capture myopathy, as a result of increased muscle cell permeability or cell damage [42, 65, 66]. Similarly, a gunshot wound might cause increased enzyme activity. Due to these factors, it is essential to consider the method of capture of the animals and how and when the blood is taken for analysis of biochemical composition [67]. However, it should be emphasized that both the endocrine and metabolic changes may have occurred simultaneously in response to the stress of being stalked, the pain of being shot, and the tissue damages associated with death.

The correlation between carcass mass (CM) and ALP in female deer is also noteworthy. It is well-known that higher levels of ALP are observed in young growing animals, which are mainly dependent on bone isoform, that exceed 50–60% of the entire amount of alkaline phosphatase [68]. Thus, two explanations for this result are possible: the female roe deer were younger than males, and it is highly probable that most of them were pregnant [69]. It is possible that the studied individuals could have been pregnant, but we are not sure, because in roe deer, latent pregnancy is usually observed in its initial period [70].

### Animal age vs. cortisol level

The studies conducted so far have not confirmed a relationship between CORT level and the age of cervids [15, 26, 33–36]. However, studies on farm fallow deer (*Dama dama*) have shown that older males have higher blood CORT levels, similar to these animals, with large changes in CORT level between analyzed periods and lower weight gain [32], which is confirmed by studies on wild roe deer. In addition, the specificity of the species and their living environment may affect CORT levels and thus the strength of the endocrine response [17, 45], which may explain the discrepancies in the results obtained by other authors.

### Limitations

The analysis of blood samples from different hunting seasons and the association of the sex of the hunted animal with the hunting season limited the conclusions that can be drawn from our data. Additional research will be needed to provide a more complete context, especially given the complexity of the animals’ stress reactions to stalking hunts.

## Conclusions

To conclude, high mean CORT value in males during the summer period were most likely due to the mating season and the associated characteristic sexual behavior of the animals. The increase in CORT in the examined animals were associated with HDL cholesterol, while correlated with lower total cholesterol, lactate dehydrogenase, and uric acid in all the researched roe deer. High levels of lactate dehydrogenase may have been caused by tissue rupture after the shot. The level of the CORT increased with the animals’ age, and increased carcass mass was linked with decreased alkaline phosphatase in females. Two of the stronger predictors of CORT levels in roe deer were age and uric acid concentrations. However, the conclusions need to be extended by further studies.

## Methods

### Study design

Roe deer were harvested during the hunting period in accordance with the principles of population and individual selection of game animals in Poland (Hunting Law, Annex to Resolution No. 57/2005 of February 22, 2005) during stalking hunts in the Lubartów forest district, central Poland (51 ° 27 ‘N, 22 ° 29’ E). The region has 24.9% forest cover, 49% of the area is coniferous, and 38% is mixed forests with deciduous species [71].

### Sampling

Blood samples were collected immediately after shooting from 16 males in August and 19 females in November, due to the fact that in Poland, roe deer are hunted during two periods only: males from May 11th to the end of September and females from September 1st to January 15th (Journal of Laws No. 43, item 488). Blood was collected from the jugular vein (*vena jugularis externa*) into 10 mL vacuum tubes with EDTA anticoagulant (BD Vacutainer, No. ref. 367,525), which cooled (4–8 °C) within 15 min of collection. Blood samples contaminated by rumen, stomach, or intestinal contents were discarded. The plasma for analysis was obtained by centrifugation of whole blood at 3000 rpm for 10 min in an MPW-350R laboratory centrifuge (MPW Medical Instruments, Warsaw, Poland) at a temperature of 4 °C. Centrifuged plasma was frozen at -25 °C until testing. CORT level was determined by enzyme immunoassay (Immulite 2000 Cortisol, Siemens, UK) according to the manufacturer’s recommended protocol. Selected biochemical parameters (total cholesterol (TCHOL), HDL cholesterol (HDLC), triglycerides (TRIG), lactate dehydrogenase (LHD), urea (UREA), alanine aminotransferase (ALT), uric acid (URIC), alkaline phosphatase (ALP), total protein (TP), and albumin (HSA) were determined with an automated spectrophotometric system (Chemical Autoanalyzer BS-120, Mindray, Shenzhen, China).

The age (A) of the animals was determined posthumously by their dentition using the Eidelman method. This involves assessing the dentin layers deposited in the canal of the incisors of the first I1 pair, and the characteristic features indicate the stage of development and permanent replacement of deciduous teeth [72]. Carcass mass (CM) (without guts) was determined after the animal was shot and after evisceration at a game collection point.

### Statistical analysis

Statistical analyses were performed using the Statistica 9.1 package. The results are expressed as the mean value and standard deviation of the variables. The level of CORT with the level of biochemical parameters for each separate group was then compared using Spearman’s rank order. Then, using the same method, correlations between the carcass mass of harvested animals and the CORT level and biochemical parameters were calculated. Equivalent calculations were used for the relationship between the age of roe deer and the CORT levels and blood chemistry values. The effect of animal age and uric acid concentration on CORT levels was assessed by multivariate linear regression analysis. The results of all correlations were estimated at a significance level of *p* < 0.05. Due to bias related to the period of sampling, we abstained from statistical significance tests comparing data of males versus females.

## Data Availability

The datasets used and analysed during the current study available from the corresponding author on reasonable request.

## Abbreviations

CORT: cortisol
TCHOL: total cholesterol
HDLC: cholesterol HDL
TRIG: triglycerides
LDH: lactate dehydrogenase
UREA: urea
ALT: alanine aminotransferase
URIC: uric acid
ALP: alkaline phosphatase
TP: total protein
HSA: albumin
A: age
CM: carcass mass
